# Intelligent Dermatologist Tool for Classifying Multiple Skin Cancer Subtypes by Incorporating Manifold Radiomics Features Categories

**DOI:** 10.1155/2021/7192016

**Published:** 2021-09-15

**Authors:** Omneya Attallah, Maha Sharkas

**Affiliations:** Department of Electronics and Communications Engineering, College of Engineering and Technology, Arab Academy for Science,Technology and Maritime Transport, Alexandria 1029, Egypt

## Abstract

The rates of skin cancer (SC) are rising every year and becoming a critical health issue worldwide. SC's early and accurate diagnosis is the key procedure to reduce these rates and improve survivability. However, the manual diagnosis is exhausting, complicated, expensive, prone to diagnostic error, and highly dependent on the dermatologist's experience and abilities. Thus, there is a vital need to create automated dermatologist tools that are capable of accurately classifying SC subclasses. Recently, artificial intelligence (AI) techniques including machine learning (ML) and deep learning (DL) have verified the success of computer-assisted dermatologist tools in the automatic diagnosis and detection of SC diseases. Previous AI-based dermatologist tools are based on features which are either high-level features based on DL methods or low-level features based on handcrafted operations. Most of them were constructed for binary classification of SC. This study proposes an intelligent dermatologist tool to accurately diagnose multiple skin lesions automatically. This tool incorporates manifold radiomics features categories involving high-level features such as ResNet-50, DenseNet-201, and DarkNet-53 and low-level features including discrete wavelet transform (DWT) and local binary pattern (LBP). The results of the proposed intelligent tool prove that merging manifold features of different categories has a high influence on the classification accuracy. Moreover, these results are superior to those obtained by other related AI-based dermatologist tools. Therefore, the proposed intelligent tool can be used by dermatologists to help them in the accurate diagnosis of the SC subcategory. It can also overcome manual diagnosis limitations, reduce the rates of infection, and enhance survival rates.

## 1. Introduction

The World Health Organization (WHO) has declared that cancer is the foremost cause of death globally. It estimates that the number of individuals identified with cancer would be twice over a subsequent couple of decades [1]. Among cancer types, skin cancer (SC) is considered one of the most common deadly tumors among both women and men populations with almost 9% of them diagnosed with SC in the United States [2]. Throughout the last few decades, countries such as Canada and Australia experienced a huge increase in the number of patients diagnosed with SC [3–5]. Moreover, in Brazil, based on the Brazilian Cancer Institute (INCA), 33% of the people affected by cancer are due to SC [6]. The rates of death and SC infection are still growing. These rates can be decreased if cancer is detected and cured during its initial stages. Primary detection of SC is the keystone to enhancing outcomes and is associated with great improvement in survival rates. Nevertheless, if the disease is progressed ahead of the skin, the survival rates become poor [7].

SC happens when skin cells are harmed and injured, for instance, by overexposure to the sun's ultraviolet radiations. SC can be classified into two main categories: melanocytic and nonmelanocytic lesions. The former category involves melanoma and nevus SC subtypes which occur in malignant and benign forms whereas nonmelanocytic lesions include basal cell and squamous cell carcinoma (SCC) which also appear in malignant and benign types. Actinic keratosis (ak) is the primary sort of SCC. Furthermore, vascular, benign keratosis, and dermatofibroma are recognized as nonmelanocytic benign lesions [8, 9]. In the present medical routine, traditional methods to diagnose and detect SC subtypes involve manual screening and visual examination. These procedures are exhausting, complicated, prone to diagnostic error, and highly dependent on the dermatologist's experience and abilities [10]. The reason for misdiagnosis is the complex patterns of skin lesions located in images [8]. Moreover, to analyze, clarify, and interpret skin lesions, the pixels of these lesions should be recognized explicitly which is hard due to several reasons [11]. First, skin lesions usually suppress hair, oils, and blood vessels that disturb the segmentation process. Furthermore, the low contrast among the lesion and the surrounding regions presents challenges in the accurate segmentation of the lesion. Lastly, these lesions commonly have distinct shapes, dimensions, and colors which increase the difficulty in precisely classifying lesions subtypes. These reasons lead to the massive need for automated intelligent systems for skin lesions analysis to overcome the above-mentioned challenges [12].

Recently, artificial intelligence- (AI-) based assistant systems have offered solutions to revolutionize medicine and health care. AI techniques have shown impressive outcomes in numerous medical fields including breast cancer diagnosis [13, 14], brain tumors [15, 16], gastrointestinal diseases [17], lung diseases [18], and heart complications [19–22]. They also revealed remarkable success in healthcare applications such as telerehabilitation [23], health monitoring [24], and assisting people with disabilities [25, 26]. Furthermore, the latest surveys [10, 27, 28] have proven the achievement of AI-based dermatologist tools in the automatic diagnosis and detection of SC diseases. These automated systems can assist and support clinicians in the fast and accurate decisions regarding the SC subtype, thus avoiding the challenges of the manual diagnosis. They can also offer a user-friendly atmosphere for nonskilled dermatologists. Moreover, they may provide a second opinion which leads to a more confident decision [29].

Radiomics is an evolving field in medical image quantitative analysis [30]. It is also known as quantitative image features. Radiomics associates the large number of significant features extracted from medical images to the biological or clinical endpoints [31]. The integration of radiomics and AI techniques has facilitated the accurate diagnosis of cancer types [32]. This is because radiomics can determine texture and other fundamental components of the tumor from medical images which help the AI methods to perform well and achieve accurate classification or diagnostic results [33]. This paper proposes an intelligent dermatologist tool for the automatic classification of several SC subtypes using an integration of AI and radiomics feature extraction techniques [34]. The motivation behind this work and the novelty of the proposed tool is discussed in the next section. The details of the proposed intelligent tool are illustrated in the methods sections.

The paper is arranged as follows.  Section 2 includes background regarding AI-enabled tools for SC diagnosis.  Section 3 involves the dataset description, methods of deep learning, and the proposed intelligent tool.  Section 4 illustrates the evaluation metrics.  Section 5 presents and discusses the results of the proposed tool and Section 5 concludes the paper.

## 2. Background on Artificial Intelligence in Skin Cancer Diagnosis

Throughout the past years, several automated tools have been introduced for SC detection and diagnosis. These tools can be classified into two classes, conventional and deep learning- (DL-) based methods. The former methods are based on traditional machine learning which includes image preprocessing, image segmentation, and feature extraction that mine low-level radiomics features based on handcrafted approaches. Monica et al. [35] proposed an automated system based on low-level radiomics feature extraction methods such as grey level covariance matrix (GLCM) and some statistical features to learn an SVM classifier to classify 8 subclasses of SC reaching an accuracy of 96.25%. Likewise, Arora et al. [36] fused several low-level features using bag of features (BoF) with SURF features to classify skin images into cancerous and noncancerous. The authors classified images using an SVM classifier and obtained an 85.7% accuracy. Also, Kumar et al [37] implemented a system for differentiating cancerous and noncancerous lesions of the skin using low-level features. First, the authors preprocessed images using a median filter. Then, they segmented the lesions using the fuzzy-C-means clustering approach. Next, they extracted textural features as GLCM and local binary pattern (LBP) as well as color features. Finally, an artificial neural network was trained using the differential evaluation algorithm to classify skin lesions reaching an accuracy of 97.7%.

On the other hand, DL-based techniques are the most recent branch of machine learning techniques that are commonly used in image processing. This is due to their great capacity in diagnosing several diseases from images even without preprocessing, segmentation, and feature extraction. They can also be used as feature extractors to extract high-level radiomics features from medical images [38–40] to be used in the classification process. Rodrigues et al. [41] designed an automated system based on DL and the Internet of things (IoT) to assist doctors in distinguishing between nevi and melanoma skin cancer subclasses. The authors utilized VGG, Inception, ResNet, Inception-ResNet, Xception, MobileNet, DenseNet, and NASNet convolutional neural networks (CNNs) as feature extractors. These high-level features were used separately to construct and train numerous classifiers. The highest performance (accuracy of 96.805%) was attained using the deep radiomics features of the DenseNet-201 and k-nearest neighbor (KNN) classifier. Similarly, Khamparia et al. [42] proposed a framework that can remotely classify skin tumors into malignant and benign using DL techniques. The authors extracted high-level deep features from four CNNs, including ResNet-50, VGG-19, Inception, and SqueezeNet using transfer learning (TL). Next, these features were utilized as inputs to the fully connected layer of a CNN for classification using dense and max-pooling operation attaining a maximum accuracy of 99.6%. Khan et al. [43] presented a novel framework for diagnosing SC subclasses. The framework consists of two main stages: segmentation and classification. In the segmentation stage, a mask recurrent CNN (R-CNN) was employed based on ResNet-50 and the feature pyramid network. Afterward, in the classification stage, a 24-layer based CNN was constructed which employs the Softmax activation function for classification. The accuracy achieved was 86.5%. Later, Khan et al. [44] preprocessed images using decorrelation deformation algorithm, and then employed mask-R-CNN for segmenting skin lesions from these images. Next, deep features from the pooling and fully connected layers of DenseNet are extracted and combined. Afterward, optimal features were selected using entropy-controlled least square SVM. The accuracy attained was 88.5%.

Alternatively, some authors combined several high-level deep features; for example, in [45], the authors mined high-level features from pretrained AlexNet and VGG-16. Afterward, these features are combined in a concatenation method and reduced using principal component analysis. Finally, these reduced features were used to learn several classifiers to classify skin tumors into malignant and benign. The Bagged tree classifier obtained the highest accuracy of 98.71%. Similarly, To˘gaçar et al. [46] introduced an intelligent system to differentiate malignant and benign skin tumors. Initially, images were reconstructed using an autoencoder and then used to train MobileNet. The original images are used to train another MobileNet. The high-level features extracted from the two MobileNet are combined, and a spiking neural network (SNN) is employed to perform classification reaching a 95.27% accuracy. Conversely, the authors of [47] extracted low-level radiomics features based on textural analysis such as GLCM and LBP features and then reduced these features using principal component analysis (PCA). Afterward, the reduced features are used to train several individual classifiers to classify malignant and benign skin lesions. Parallelly, the authors extracted high-level features from a VGG-19 and a customized CNN to classify images into malignant and benign using individual classifiers. Finally, the predictions attained using both levels of features are merged using a voting ensemble classifier reaching a 97.5% accuracy.

The aforementioned techniques have several drawbacks. First, most of them were constructed for binary classification problems such as differentiating between benign and malignant, cancerous and noncancerous, or two skin lesions subclasses. A few of them have classified several subtypes of cancer discussed earlier. The majority of them are either based on the low level or high level of features except for [47] that have fused both levels to perform binary classification. These shortcomings have motivated us to propose a new intelligent dermatologist tool to classify seven skin cancer categories. The proposed tool examines the influence of combining two low-level radiomics features. It also studies the effect of merging several high-level deep features. Finally, it investigates the impact of fusing manifold low-level and high-level features.

## 3. Methods and Materials

### 3.1. Feature Extraction Methods

#### 3.1.1. High-Level Radiomics Features Based on Deep Learning Techniques

ResNet is one of the most potent CNNs that are commonly used in the medical field. It received a prominent place in ILSVRC and COCO 2015 competition [48]. It has high capabilities to converge effectively with adequate computation time despite the expanding number of layers. This superiority is due to its new construction introduced by He et al. [48] that entirely relies on the deep residual block. This block embeds shorter paths along the conventional deep CNN to exclude some layers during the training phase which leads to great acceleration in the convergence process [18]. The number of deep layers used in the pretrained ResNet employed in the paper is 50.

DenseNet: several research articles have stated that deep networks may be considerably deeper, accurate, and time-cost effective when created with shorter ties including layers close to the input and output. Thus, the Dense Convolutional Network (DenseNet) was implemented by Huang et al. [49] depending on the aforementioned short links. DenseNet ties all the layers to each other in a feed-forward practice where feature maps are inputs to the subsequent layer while the feature maps of the current layer are supplied to the whole succeeding layers. The DenseNet CNN included in this study has 201 deep layers.

DarkNet was initially implemented by Redmon and Farhadi [50] in 2017. It extremely depends on YOLO-V2. It has a cascaded series of convolutional layers having sizes of 1 × 1 and 3 × 3 which are doubled after each pooling process. DarkNet employs a global average pooling layer to lower the feature presentation between the 3×3 convolutional layers. The number of deep layers involved in the DarkNet used in this study is 53.

#### 3.1.2. Low-Level Features Based on Handcrafted Techniques

Discrete wavelet transform (DWT) applies orthogonal basis functions termed “wavelets” to analyze input data [51]. For 1D input data as the deep radiomics features mined in the earlier phases, the DWT procedure is accomplished through convolving the input features with a low and high pass filter [52]. After that, a reduction process is accomplished by downsampling the output data by 2 [53]. Subsequently, two clusters of coefficients are produced called the approximation coefficients CA_1_ and detail coefficients CD_1_ [54].

Local binary pattern (LBP) was proposed by Ojala et al. [55] as a feature extractor approach that determines the local demonstrations and information from pixels. It simply transforms an image into a set of local textures. LBP gives a binary label to each pixel value in an image according to a specific threshold calculated from the neighbor pixel values around the center pixel.

### 3.2. Dataset

**Figure 1:**
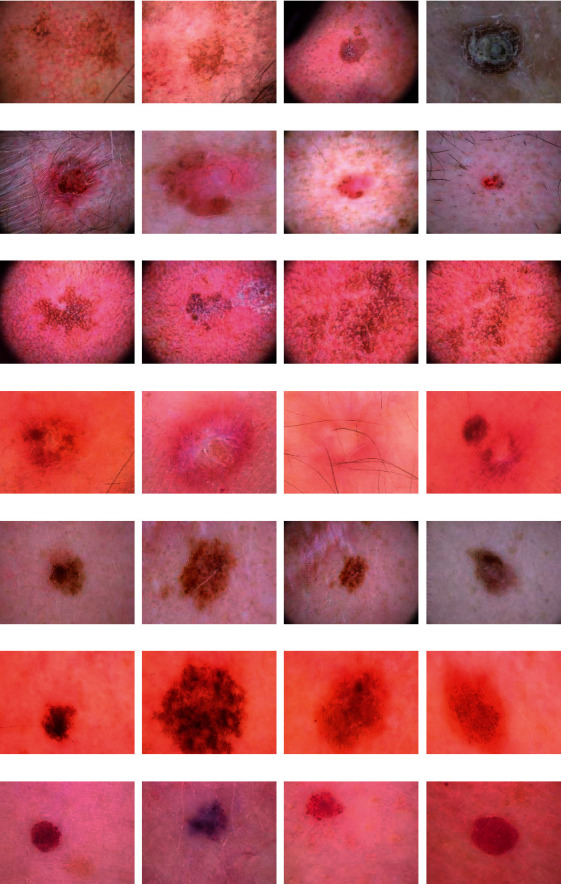
Samples of HAM10000 dataset: (a) ak, (b) bcc, (c) blk, (d) df, (e) mel, (f) nv, and (g) vasc SC class.

The dataset used in this work is called HAM10000 [56]. This dataset contains images of seven subclasses of SC including melanoma (mel), nevus (nv), basal cell carcinoma (bcc), actinic keratosis (ak), vascular (vasc), benign keratosis (bkl), and dermatofibroma (df). The HAM10000 dataset consists of 10,008 images dermoscopic photos. Among these images 514 are bcc, 327 are ak, 6705 are nv, 1095 are bkl, 1110 are mel, 115 are df, and 142 are vasc skin lesion subtypes. Samples of these images are shown in Figure 1.

### 3.3. Proposed Intelligent Dermatologist Tool

**Figure 2:**
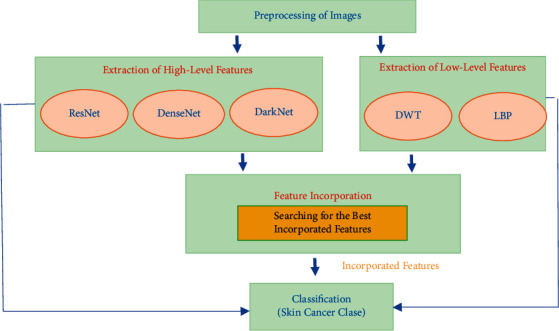
The block diagram of the proposed intelligent dermatologist tool.

The proposed intelligent tool consists of four steps involving preprocessing of the dermoscopic photos, feature mining, feature incorporation and selection, and classification steps. Initially, photos are resized and augmented. Then, in the feature mining step, low-level features are extracted from two traditional feature extractions. Also, high-level features are mined using three DL techniques. Afterward, features of different levels are integrated and examined and then reduced in the feature incorporation and selection step. Finally, three support vector machine (SVM) classifiers are utilized to classify multiple SC subclasses. The block diagram of the proposed intelligent dermatologist tool is shown in Figure 2.

#### 3.3.1. Preprocessing of Dermoscopic Images

The dermoscopic images of the HAM10000 dataset are of different sizes; therefore, they are all resized to the corresponding dimension of each of the CNNs DL techniques used in this work (224 × 224 × 3 for ResNet-50 and DenseNet-201, and 256 × 256 × 3 for DarkNet-53). Furthermore, as noticed in the dataset section, the number of photos in each class of the dataset is unbalanced; therefore, we used several augmentation techniques to balance the dataset. These augmentation techniques include shearing, rotation, and top and bottom hat filtering. The number of images after augmentation is 1028 for bcc, 981 for ak, 1050 for nv, 1095 for bkl, 1110 for mel, 920 for df, and 994 for vasc.

#### 3.3.2. Feature Mining

In this step, two categoriesof radiomics features are mined consisting of low level and high levelfeatures. In the low-level features, two handcrafted feature extraction methods including LBP [57] and DWT [58] are used. These techniques are based on texture analysis which frequently produces sufficient classification performance, particularly when merged [59]. In the DWT, 3 decomposition levels with Daubechies 4 (db-4) mother wavelet are made. The coefficients of approximation coefficients CA_3_ and three detail coefficients CD_3_ are considered as low-level features.

On the other hand, the high-level features include features extracted from three DL approaches. These techniques are the ResNet-50, DenseNet-201, and DarkNet-53 CNNs. To mine these features, initially, TL [60] is performed on the three deep pretrained CNNs learned with the ImageNet dataset to be capable of classifying the seven skin lesion categories. Afterward, few parameters are adjusted for each CNN. Next, the three CNNs are trained with images of the HAM10000 dataset after being resized and augmented. Lastly, high-level features are extracted from the last average pooling layer of the three CNNs. The dimensions of the high-level and low-level features are shown in Table 1.

To reproduce the high-level features, first, some parameters of the three CNNs should be adjusted such as the learning rate (0.003), number of epochs to 30, validation frequency to 20, and min-batch size to 4. Afterward, TL is employed to use the pretrained CNNs (previously trained on the ImageNet dataset) and change the number of output layers to seven. Next, the three CNNs are trained with the HAM10000 dataset using stochastic gradient descent with a momentum algorithm. Finally, TL is used to extract the high-level features from the latest average pooling layer of the three CNNs. Some features comply with the 174 standards of image biomarker standardization initiative (IBSI) [61, 62] while others are not.  Table S1 in the supplementary material discusses the compliance/noncompliance of these features.

#### 3.3.3. Feature Incorporation and Selection

The feature incorporation step is accomplished in three phases. In the first phase, the low-level features extracted in the feature mining stage are integrated using a concatenated procedure. In the second phase, high-level features are fused in a concatenated manner. Additionally, in the third phase, each combination of low- and high-level feature sets is combined to determine the influence of incorporating manifold feature categories and select the integrated manifold feature combination which impacts the classification performance. After accomplishing the incorporation phases, the integrated features set that accomplished the high impact on the classification performance undergo a feature selection stage. Feature selection is done to reduce the huge dimension of fused features. Minimum redundancy maximum relevance (mRMR) feature selection procedure [63] is used in this step.

#### 3.3.4. Classification

In the classification step, the well-known SVM classifier is used to classify the seven subclasses of SC. The kernel functions employed in the classification process are linear, cubic, and quadratic. The 5-fold cross-validation (CV) method is utilized to validate the classification outcomes of the proposed dermatologist tool. In the CV procedure, the dataset is initially split into 5 equal folds. Afterward, 4 folds of them are employed in the training process of the SVM classifiers, where the 5^th^ fold is used for testing. This process is repeated 5 times where at each time the SVM classifiers are trained with different 4 training folds and the 5^th^ is used for testing. Several performance metrics that will be mentioned in the next sections are calculated for each testing fold and averaged for the 5 folds.

## 4. Metrics of Performance

Some metrics are used to measure the performance of the proposed intelligent dermatologist tool, including classification accuracy (CA), F1-score, sensitivity, precision, and specificity [16]. Formulas (1)–(5) are used to determine these metrics:

(1) $$\begin{array}{c} \text{accuracy}=\;\frac{TP+TN}{TP+TN+FP+FN}\;, \end{array}$$

(2) $$\begin{array}{c} \text{sensitivity}=\frac{\text{TP}}{\text{TP}+\text{FN}}, \end{array}$$

(3) $$\begin{array}{c} \text{specificity}=\frac{\text{TN}}{\text{TN}+\text{FP}}, \end{array}$$

(4) $$\begin{array}{c} \text{precision }=\;\frac{\text{TP}}{\text{TP}+\text{FP}}, \end{array}$$

(5) $$\begin{array}{c} F1-\text{score}=\frac{\left(2\times \text{precision}\times \text{recall}\right)}{\text{precision}+\text{recall}}, \end{array}$$

where TP is the true positive, FN exemplifies false negative, TN represents the true negative, and FP is the and false-positive.

## 5. Results and Discussion

This section will present and discuss the results of the proposed dermatologist tool. The section will first discuss the classification results utilizing low-level features. Afterward, it will show and illustrate the classification outputs using the high-level features. Next, it will introduce and explain the classification outcomes using the integration of manifold radiomics feature categories. Finally, it will compare the results of the proposed intelligent dermatologist tool with recent related works constructed with the same dataset to verify its competence.

### 5.1. Results of Low-Level Features

**Figure 3:**
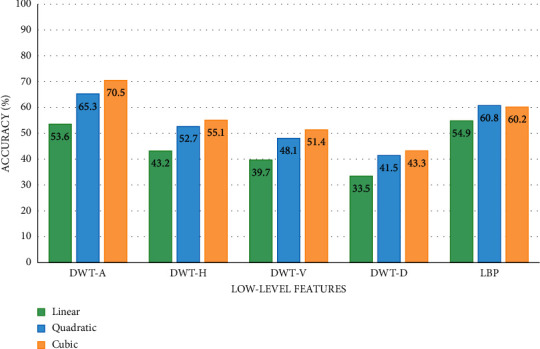
The SVM classifiers' accuracy (%) obtained using the low-level features.

The classification results of the SVM classifiers trained with low-level features including DWT and LBP are shown in Figure 3. Note that the DWT-A, DWT-H, DWT-V, and DWT-D correspond to the approximation, horizontal, vertical, and diagonal DWT coefficients, respectively. As it can be noticed from Figure 3, the SVM classifiers trained with low-level features produce classification accuracy that ranges between 33.5 and 70.5%. The highest accuracy is obtained with the cubic SVM classifier constructed using the DWT-A features. These results verify that using low-level features alone is not capable of reaching accurate results for SC classification.

### 5.2. Results of High-Level Features

**Figure 4:**
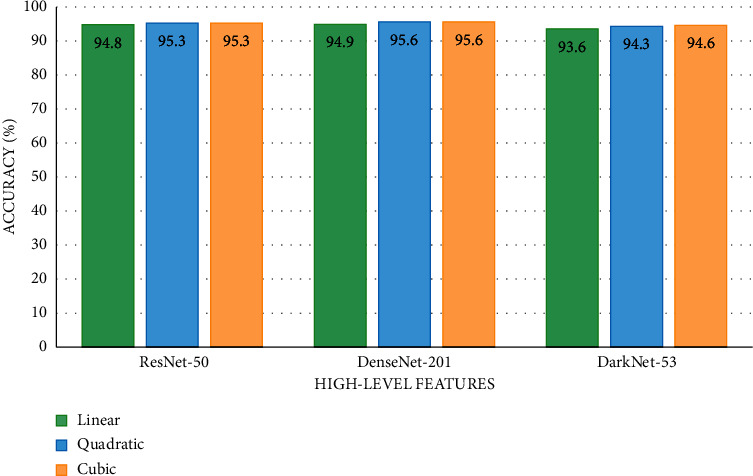
The SVM classifiers' accuracy (%) obtained using the high-level features.

The outputs of the SVM classifiers learned with high-level features of DenseNet-201, ResNet-50, and DarkNet-53 CNNs are shown in Figure 4. The maximum accuracies of 95.6%, 95.6%, and 94.9% are obtained by the cubic, quadratic, and linear SVM classifiers correspondingly trained with the high-level features of DenseNet-201. Slightly lower accuracies (95.3%, 95.3%, and 94.8%) are achieved using the same classifiers learned with ResNet-50 features. The DarkNet-53 features accomplish accuracies of 94.6%, 64.3%, and 93.65 using the cubic, quadratic, and linear SVM classifiers, respectively. Figure 4 proves that utilizing high-level features has higher classification accuracy compared to low-level features shown in Figure 3.

### 5.3. Results of Incorporating Manifold Feature Categories and Feature Selection

The classification accuracies for the SVM classifiers trained with the incorporated manifold levels features are shown in Table 2. Table 2 first illustrates the accuracy attained using each combination of high-level features with low-level features. As it is clear, the fusion of every single high-level feature with one and two low-level feature sets has improved the accuracy of classification reaching peak accuracies of 97.5%, 97.9%, and 97.9% (linear, quadratic, and cubic SVM correspondingly) using the incorporation of DenseNet-201+ DWT-A + LBP features. These accuracies are higher than those attained using either the individual high-level features or single low-level features shown in Figures 3 and 4.

Next, Table 2 discusses the results of fusing every two high-level features as well as combining every two high-level feature sets with low-level features. Table 2 verifies that combining two high-level features has a positive impact on the accuracy as it increases to reach 97.9%, 98.1%, and 98% (linear, quadratic, and cubic SVM, respectively) using DenseNet-201 + DarkNet-53 high-level features. Moreover, when merging two high-level features with two low-level features, the classification accuracies of the SVM classifiers are enhanced to reach maximum accuracies in this scenario of 98.2%, 98.6%, and 98.5% utilizing the combined features of ResNet-50 + DenseNet-201 + DWT-A + LBP which are higher than those achieved when combining one high-level feature set with low-level features.

**Figure 5:**
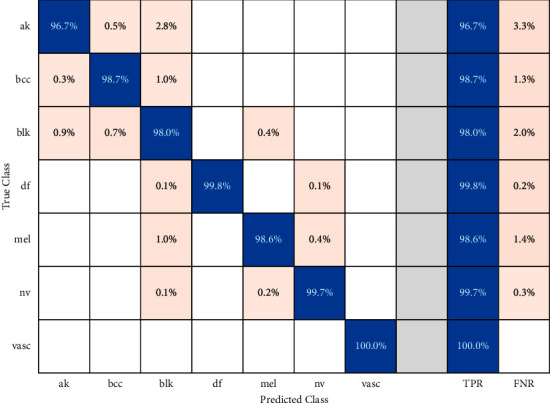
Confusion matrix for cubic SVM classifier trained with ResNet-50 +DenseNet-201 + DarkNet-53 + DWT-A + LBP features.

Finally, Table 2 displays the classification accuracies of fusing the three high-level features along with integrating the three high-levels features with low-levels features. Table 2 proves that incorporating manifold features of different categories has a high impact on classification accuracy. This is obvious as when merging the three high-levels features of ResNet-50 + DenseNet-201+ DarkNet-53 with the low-level features of DWT-A + LBP, the accuracy is boosted to 98.7%, 99%, and 99% (linear, quadratic, and cubic SVM, respectively). This improvement in the classification accuracy indicates the capacity of the proposed intelligent dermatologist tool in classifying the subclasses of skin cancer.  Figure 5 shows the confusion matrix for the cubic SVM classifier trained with the manifold features of ResNet-50 + DenseNet-201+ DarkNet-53 + DWT-A + LBP.

**Figure 6:**
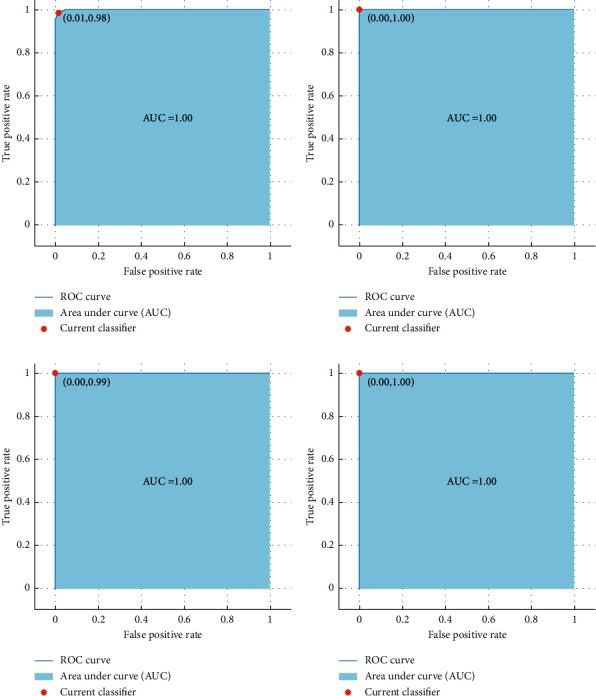
ROC curves along with the AUC for quadratic SVM classifier, (a) blk is the positive class, (b) df is the positive class, (c) nv is the positive class, and (d) vasc is the positive class.

The performance metrics including the sensitivity, specificity, precision, and F1-score for the cubic SVM classifier trained with ResNet-50 +DenseNet-201 + DarkNet-53 + DWT-A + LBP features are shown in Table 3. Table 3 shows that the mean specificity, sensitivity, precision, and F1-score for the seven classes of SC are 0.9969, 0.9854, 0.9884, and 0.988. These results verify that the proposed dermatologist tool is reliable. This is because, as stated in [64–66], for any medical system to be reliable, the precision and specificity must be more than 0.95 and sensitivity should exceed 0.8. The receiving operating characteristics (ROC) curves along with the area under the curve (AUC) are displayed in Figure 6.

**Figure 7:**
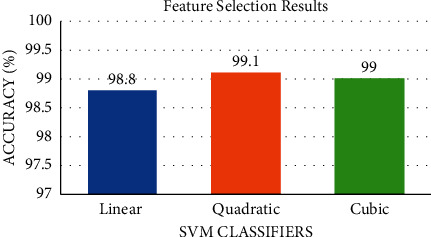
The classification accuracy after the mRMR feature selection procedure for the three SVM classifiers.

**Figure 8:**
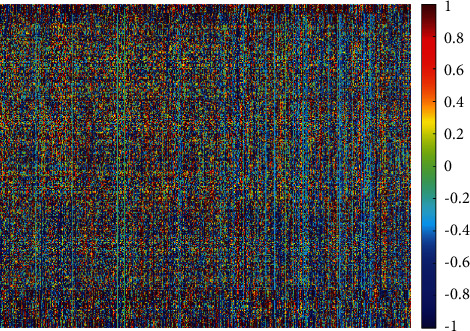
Heat map analysis of the selected radiomics features.

The results after using the mRMR feature selection approach are shown in Figure 7. Note that the classification accuracy for both quadratic and linear SVM has increased to 99.1% and 98.8%, respectively, whereas, for the cubic SVM, the accuracy is the same (99%). The mRMR feature selection procedure has reduced the number of features to 2500 which is lower than the 6495 of the combined manifold features of ResNet-50 + DenseNet-201+ DarkNet-53 + DWT-A + LBP.  Figure 8 shows the heat map analysis of the selected radiomics features.

### 5.4. Comparing the Performance of the Proposed Tool with Related Works

To verify the competence of the proposed intelligent dermatologist tool, its performance is compared with recent related studies based on the HAM1000 dataset. This comparison is shown in Table 4. It is obvious from Table 4 that the proposed tool has a superior performance compared to other related works since the accuracy, sensitivity, specificity, and F1 score achieved using the proposed tool are 99%, 98.54%, 99.69%, 98.84%, and 98.83% which are greater than all other studies. This outperformance is because the proposed intelligent dermatologist tool is based on incorporating manifold features categories. It first examined the use of three individual high-level features and then two low-level handcrafted features. Next, it investigated the influence of incorporating several high- and low-level features and searched for the best-integrated manifold features. The results of the proposed tool have shown that merging manifold features of different categories have a great impact on classification accuracy. This is not the case in other related studies shown in Table 4 as they are based on either low-level features or high-level features. Most of them employed individual features sets and did not examine the influence of feature fusion.

Early detection of SC is very important to prevent it from progression. It can also help in choosing appropriate treatments and follow-up plans and decreasing death rates. This study proposed an intelligent tool for the automatic classification of lesion types. The results achieved using the proposed intelligent tool are promising. They verify that the proposed tool is an effective method that can be used in clinical practice. In this common sense, the key privilege of the proposed tool is its accessibility which means that it can be used in several regions easily especially those which suffer from the lack of skilled dermatologists. Besides, this tool will enable dermatologists to automatically diagnose the SC subclass and avoid challenges they face during manual examinations due to the complex patterns of skin lesions located in SC images [8]. It will also ease and fasten the diagnosis procedure compared to manual diagnosis. Moreover, the accurate classification of the lesion using the proposed tool will prevent patients diagnosed with a noncancerous lesion from the excess hospital visits, as normal medication can cure them without the need for exposure to radiation or chemotherapy. On the other hand, the tool can accurately diagnose patients with the specific SC category which helps doctors to select the suitable treatment procedure. Several studies have studied the use of individual feature extraction methods including traditional low-level features and high-level features based on deep learning to diagnose SC; however, the fusion among these features is of great importance, as the results of the proposed tool showed that integrating these features can enhance the performance. The results of the proposed tool prove that this tool adds value to the healthcare division. This is because the tool can diagnose the SC category more accurately than those methods used in the literature.

## 6. Conclusion

Skin cancer (SC) is one of the widespread malignant tumors among human populations. The increasing rate of infection of this type of cancer can be reduced if accurately diagnosed and treated during its initial stages. This paper proposed a dermatologist tool based on AI methods and manifold radiomics features categories to enable doctors to accurately diagnose the SC subtype. This could facilitate choosing the appropriate follow-up and treatments plans. The proposed intelligent tool is based on several deep learning and machine learning techniques. It incorporates manifold radiomics features categories including three high-level features of ResNet-50, DenseNet-201, and DarkNet-53 and two low-level radiomics features of DWT and LBP. This study proved that integrating both levels of radiomics features boosted the performance of the dermatologist tool compared to using either high-level or low-level features alone. The performance of the intelligent dermatologist tool was compared with related AI-based dermatologist tools and this comparison verified the superiority of the proposed tool over other tools; thus the proposed intelligent tool can be used to assist dermatologists in the accurate diagnosis of the subcategory of SC and avoid the complications of manual diagnosis. Upcoming work will consider using more deep learning techniques, other radiomics techniques, segmentation methods, and applying other integration techniques. The main limitation of this tool is using the 5-fold cross-validation method for validating the performance; however, cross-center validation using other datasets is required. Therefore, future work will consider using another dataset for cross-center validation.

## Figures and Tables

**Table 1:** The size of low-level and high-level features.

Feature type	Size
*Low-level features*	
DWT-A	1444
DWT-V	1444
DWT-H	1444
DWT-D	1444
LBP	59

*High-level features*	
ResNet-50	2048
DenseNet-201	1920
DarkNet-53	1024

**Table 2:** Classification accuracy of incorporating manifold feature levels.

Incorporated manifold feature sets	Linear	Quadratic	Cubic
*Single high-level features incorporated with low-level features*			
ResNet-50 + DWT-A	95.7	96.1	96.2
ResNet-50 + LBP	96.1	96.4	96.4
ResNet-50 + LBP + DWT-A	96.5	96.9	96.8
DarkNet-53 + DWT-A	94.6	94.9	95.1
DarkNet-53 + LBP	95.8	96	96.1
DarkNet-53 + DWT-A + LBP	95.7	95.9	96
DenseNet-201 + DWT-A	96.5	97.1	97.2
DenseNet-201 + LBP	97.1	97.5	97.4
DenseNet-201 + DWT-A + LBP	97.5	97.9	97.9

*Two high-level feature sets incorporated*			
ResNet-50 + DarkNet-53	95.9	96.3	96.6
ResNet-50 + DenseNet-201	97.7	98	98.1
DenseNet-201 + DarkNet-53	97.9	98.1	98

*Two high-level feature sets incorporated with low-level feature sets*			
ResNet-50 + DenseNet-201 + DWT-A	98.2	98.5	98.5
ResNet-50 + DenseNet-201 + LBP	97.8	98.1	98.1
ResNet-50 + DenseNet-201 + DWT-A + LBP	98.2	98.6	98.5
ResNet-50 + DarkNet-53 + DWT-A	96.4	96.9	96.9
ResNet-50 + DarkNet-53 + LBP	96.7	96.9	97
DenseNet-201 + DarkNet-53 + DWT-A	98.2	98.4	98.5
DenseNet-201 + DarkNet-53 + LBP	97.9	98.4	98.4
DenseNet-201 + DarkNet-53 + DWT-A + LBP	98.3	98.6	98.6

*Three high-level feature sets incorporated*			
ResNet-50 + DenseNet-201 + DarkNet-53	98.2	98.5	98.5

*Three high-level feature sets incorporated with low-level feature sets*			
ResNet-50 + DenseNet-201 + DarkNet-53 + DWT-A	98.6	98.8	98.8
ResNet-50 + DenseNet-201 + DarkNet-53 + LBP	98.5	98.8	98.7
ResNet-50 + DenseNet-201 + DarkNet-53 + DWT-A + LBP	98.7	99	99

**Table 3:** Performance metrics for the cubic SVM classifier trained with ResNet-50 +DenseNet-201 + DarkNet-53 + DWT-A + LBP features.

Class	Specificity	Sensitivity	Precision	F1-score
ak	0.9979	0.9674	0.98765	0.9768
bcc	0.9979	0.9874	0.9874	0.9874
blk	0.9918	0.9799	0.9555	0.9675
df	1	1	1	1
mel	0.999	0.9865	0.9946	0.9905
nv	0.9992	0.9971	0.9952	0.9962
vasc	1	1	1	1
mean	0.9969	0.9854	0.9884	0.9883

**Table 4:** Performance comparison between the proposed intelligent tool and related works based on the HAM1000 dataset.

Article	Accuracy (%)	Sensitivity	Specificity	Precision	F1-score
[67]	85.8	—	—	—	—
[43]	86.5	85.57%	—	87.01%	86.28%
[44]	88.5	—	—	88.66%	88.66%
[68]	90.72	—	—	—	—
[69]	90.67	90.2%	—	—	—
[70]	92.08	92.53%	—	93.73%	92.74
[35]	96.25	—	—	—	—
[37]	97.4	92%	90%	—	—
Proposed tool	99	98.54%	99.69%	98.84%	98.83%

## Data Availability

The dataset employed in the paper can be found in Kaggle (https://www.kaggle.com/kmader/skin-cancer-mnist-ham10000). Codes are available at the following link: https://drive.google.com/file/d/1ifD8xzUm-lxzvvLghrjbeo55uar8Xio8/view?usp=sharing.

## Supplementary Materials

Supplementary Materials Some radiomics features extracted in this study comply with the 174 standards of IBSI. Table S1 illustrates the compliance/noncompliance of these features.
