# Supplementary material for: Intelligent Dermatologist Tool for Classifying Multiple Skin Cancer Subtypes by Incorporating Manifold Radiomics Features Categories
Source: Contrast Media Mol Imaging. 2021 Sep 15;2021:7192016. doi: 10.1155/2021/7192016 (PMC8457955; doi:10.1155/2021/7192016)
Supplement: Supplementary Materials — Some radiomics features extracted in this study comply with the 174 standards of IBSI. Table S1 illustrates the compliance/noncompliance of these features. [file 7192016.f1.docx]

Table S1 IBSI compliance sheet

| **Feature Extraction Methods** | **Compliance with IBSI Standards** |
| --- | --- |
| Discrete Wavelet Transform | Complies with IBSI standards and has been used in [1],[2] |
| Local Binary Pattern | Complies with IBSI standards and has been used in [3], [4] |
| DenseNet-201, DarkNet-53, ResNet-50 | Do not comply with IBSI standards but have been used in [5],[6], [7] |

References

[1] L. Lu *et al.*, “Identifying Robust Radiomics Features for Lung Cancer by Using In-Vivo and Phantom Lung Lesions,” *Tomography*, vol. 7, no. 1, pp. 55–64, 2021.

[2] C. M. Chiesa-Estomba, O. Echaniz, E. Larruscain, J. A. Gonzalez-Garcia, J. A. Sistiaga-Suarez, and M. Graña, “Radiomics and texture analysis in laryngeal cancer. Looking for new frontiers in precision medicine through imaging analysis,” *Cancers*, vol. 11, no. 10, p. 1409, 2019.

[3] N. C. D’Amico *et al.*, “Radiomics-Based Prediction of Overall Survival in Lung Cancer Using Different Volumes-Of-Interest,” *Applied Sciences*, vol. 10, no. 18, p. 6425, 2020.

[4] L. Wu *et al.*, “Preoperative ultrasound radiomics analysis for expression of multiple molecular biomarkers in mass type of breast ductal carcinoma in situ,” *BMC Medical Imaging*, vol. 21, no. 1, pp. 1–14, 2021.

[5] J. Zhou *et al.*, “Diagnosis of benign and malignant breast lesions on DCE-MRI by using radiomics and deep learning with consideration of peritumor tissue,” *Journal of Magnetic Resonance Imaging*, vol. 51, no. 3, pp. 798–809, 2020.

[6] F. Gao *et al.*, “Hybrid network with difference degree and attention mechanism combined with radiomics (H-DARnet) for MVI prediction in HCC,” *Magnetic Resonance Imaging*, 2021.

[7] S. Modi, R. Guhathakurta, S. Praveen, S. Tyagi, and S. N. Bansod, “Detail-oriented capsule network for classification of CT scan images performing the detection of COVID-19,” *Materials Today: Proceedings*, 2021.
